# A descriptive study of female suicide deaths from 2005 to 2011 in Van city, Turkey

**DOI:** 10.1186/s12905-016-0299-1

**Published:** 2016-04-23

**Authors:** Yavuz Hekimoglu, Ipek Esen Melez, Nergis Canturk, Z. Zerrin Erkol, Mustafa Gokhan Dizdar, Gurol Canturk, Deniz Oguzhan Melez, Ziya Kir

**Affiliations:** Department of Forensic Medicine, Faculty of Medicine, Yuzuncu Yil University, Van, Turkey; Department of Forensic Medicine, Faculty of Medicine, Bezmialem Vakıf University, Istanbul, Turkey; Department of Criminalistics, Institute of Forensic Sciences, Ankara University, Ankara, Turkey; Department of Forensic Medicine, Faculty of Medicine, Abant Izzet Baysal University, Bolu, Turkey; Manisa Branch Office, Ministry of Justice Council of Forensic Medicine, Manisa, Turkey; Department of Forensic Medicine, Faculty of Medicine, Ankara University, Ankara, Turkey; Ministry of Justice Council of Forensic Medicine, Istanbul, Turkey; Department of Forensic Medicine, Faculty of Medicine, Celal Bayar University, Manisa, Turkey

**Keywords:** Forensic science, Forensic pathology, Women, Suicide, Death, Autopsy

## Abstract

**Background:**

Female suicide is an important problem not only for women but for public health in general.

**Methods:**

Autopsy reports from the Van Chief Public Prosecutor’s Office from 2005 to 2011 were reviewed retrospectively in order to analyse female deaths from suicide.

**Results:**

Sixty-six female suicide cases were recorded during 2005–2011. The mean age of the cases was 22.64 (sd = 10.09) years, and nine were below the age of 15. The most common method of suicide was hanging (44 cases, 66.7 %). Five (7.6 %) of the cases were under treatment for depression, and 12 (18.2 %) cases had a previous suicide attempt.

**Conclusions:**

Cultural suppression of females and prevention of their socialization in enclave societies are risk factors for female suicides. The number of female suicide attempts, especially recurring attempts, should be reduced via psychiatric scanning, follow-up sessions and therapy for high-risk individuals.

## Background

Mostly comprising high, rugged mountain areas, Van is a province in the Eastern Anatolia region of Turkey with a population of 1,035,418 and an agriculture-based economy; a patriarchal lifestyle predominates [1]. Eastern culture prevails over the social life and status of women in the society.

Suicide is a complex psychopathological phenomenon involving biological, genetic and environmental risk factors [2, 3]. According to the literature, completed suicide is more frequent among males than females. However, different results have been obtained from different regions [4–6]. Additionally, in Turkey, as a developing country, completed suicides are more commonly seen among males when compared to females. However, female suicides seem to be more frequent than male suicides in Van [7].

When an officially married man marries another partner in a religious ceremony, it is referred to in Turkish as taking a *kuma* (‘fellow wife’). An exchange marriage between two families in which a daughter and a son in one family marries a daughter and a son in the other family is called a *berdel* (‘bride exchange’). Both the *kuma* and *berdel* marriage types are seen frequently in eastern region provinces of Turkey and are prone to cause problems for the women when executed without their approval [8]. Having a fellow wife through exchange of brides is common in Van. When the women involved do not consent, the addition of a fellow wife negatively affects the officially married first wife and the psychological status of both women, which can lead to unrest, arguments and violence. Moreover, in most cases, bridal exchange is arranged by families without the consent of the daughter, and it is very unlikely that she will be happy and at ease in such a marriage. Therefore, both marriage types may present a risk for suicide attempts among women.

The Van Women’s Association (VAKAD) is a nongovernmental organization established in Van that works to strengthen the role of women in economic, social, personal, cultural and political areas. A study conducted in 2006 by this organization evaluated the causes driving women to suicide, examining not only the chief public prosecutor’s reports but also the news in local or national media about the cases. In addition, the researchers interviewed family members as well as women who had survived suicide attempts. The study identified the following causes of female suicide: the exchange of brides and fellow wives in marriage; forcing girls into an unwanted marriage through religious ceremony at a young age; the high rate of marriage between relatives; forcing the productive women in the village to stay at home after immigration to the city; lack of adequate employment opportunities; difficulty in adapting to city life; exposure to more stress than in the village; facing poverty; domestic conflicts caused by financial problems due to very low wages for the men; difficult living conditions; hidden violence within the tribal and feudal structure; being unable to avoid various types of violence due to lack of knowledge about their legal rights and inadequate support from their families; inability to exercise civil rights, such as getting divorced, marrying the man of their choice, or getting a job; impaired mental health; not having a son or being childless instead of "altogether" it should be "alltogether".; loss of sense of trust and belonging due to abuse; the belief that they will not be protected if they report violence or abuse to legal authorities; the feeling of not being treated as an individual; and not having the right to contribute to family decisions [9].

KAMER, a women’s centre, is a non-governmental organization established to identify the local practices of a sexist system that are harmful to children and women and to develop projects to combat these practices. The Diyarbakır branch of KAMER implemented a research study in nine cities, including Van, in eastern and southeastern regions of Turkey known to have serious issues with women’s rights. A total of 13,673 women were visited, and face-to-face interviews were conducted. The study determined that 16.09 % of women were married between the ages of 10 and 14 years, 64.69 % between the ages of 15 and 19 and 16.74 % between the ages of 20 and 24 [10]. It has been reported that forced marriages at young ages constitute a risk for suicide attempts by the new brides [9].

The timing (month, season, day of the week, and time of the day) of suicides and the correlation between lunar periods and suicide are topics that have been interesting research areas [11–13].

Suicide among women is strongly correlated with violence. One study reported the following risk factors for suicide among women: violence of various types from partners, physical violence from a person other than the partner, divorce, a deteriorating relationship, being a widow, sexual abuse during childhood and having a mother who is exposed to violence from her partner [14]. The domestic violence that women are exposed to is not limited to being beaten but also includes psychological, economic and sexual violence. Domestic violence in Turkey might lead many women to commit suicide. The increased numbers of charges against tribal murders in Turkey have raised the suspicion that women might choose to commit suicide rather instead of "than risk being", it should be "than the risk of being". killed in the name of traditions [15].

In addition, mood disorders, substance abuse and prior suicide attempts are strongly associated with suicides. Factors such as family adversity and social alienation also contribute to suicide risk [16].

The goal of this descriptive study was to examine the nature and characteristics of female suicide deaths that occurred in Van, an eastern province of Turkey, including technical and social parameters; to propose solutions to the problem; and to highlight the roles of domestic violence, poor sociocultural status and marital problems in suicidal behaviour.

## Method

### Universe and phases of the research

Forensic autopsies are performed on all traumatic (deaths due to suicide, homicide and accident) and sudden unexpected deaths in Turkey [17]. In Van, autopsies are performed for all suspected suicide deaths by forensic specialists in the presence of prosecutors. In such cases, the prosecutor evaluates claims, complaints (if any), incident-scene investigation data, witness statements and autopsy findings and seeks to determine the origin of the incident.

In this study, two forensic specialists who performed the autopsies and are among the authors of the article, retrospectively reviewed a total of 1493 autopsy cases that belong to the Van Chief Public Prosecutor’s Office and that occurred between 1 January 2005 and 31 December 2011. The origin of death was determined to be suicide in 123 cases, including 66 females. These female suicide cases were included in the study because Van has serious problems concerning women’s rights due to its patriarchal, feudal social structure. Additionally, female suicide rates have been found to be higher than male suicide rates in Van, unlike most other cities in Turkey. This study was designed as an original project and not as a continuation of another study.

### The basics of the retrospective study as a research method

Autopsy reports and legal files pertaining to 66 female suicide deaths were retrospectively reviewed and evaluated with respect to age group; the year, season and month in which the incident occurred; marital status, number of children, professional and economic status, history of psychiatric illness, history of previous suicide attempts, presence of traumatic findings, scene of the incident and suicide method. The statistical analysis was performed using SPSS 16.0 and chi-square tests. Statistical significance was inferred at *p* < 0.05. Because it was impossible to obtain medical records from hospitals, the information on psychiatric treatment and previous suicide attempts were obtained from the relative who identified the body and from the medical records that the relative provided to the prosecutor. All procedures in the study were performed in accordance with the ethical standards of the Bezmialem Vakıf University Ethical Committee with the official approval dated 5 February 2016 (registry no. 1919 in the database of national clinical trials). Permission to access and use the data from the Van Chief Public Prosecutor’s Office was obtained, with approval from the Chief Prosecutor.

## Results

The mean age of the cases was 22.64 (sd = 10.09) years, and the age range was 9–70. It was remarkable that nine (13.6 %) of the cases were under the age of 15. The majority of the cases (30 cases, 45.45 %) were in the 16 to 20 age group, and 30 (45.45 %) cases were children under the age of 18. Only five cases (7.6 %) were over the age of 35 (Table 1).

**Table 1:** Distribution of suicide methods according to age groups

Age Groups	Suicide Methods											
	Hanging		Firearm injury		Insecticide intoxication		Fall from a height		Firearm injury and Stabbing		Total	
	*n*	%	*n*	%	*n*	%	*n*	%	*n*	%	*n*	%
15 and below	6	9.1 %	2	3.0 %	1	1.5 %	–	–	–	–	9	13.6 %
16–20	20	30.3 %	9	13.6 %	–	–	–	–	1	1.5 %	30	45.5 %
21–25	6	9.1 %	3	4.5 %	–	–	–	–	–	–	9	13.6 %
26–30	5	7.6 %	2	3.0 %	–	–	–	–	–	–	7	10.6 %
31–35	4	6.1 %	–	–	2	3.0 %	–	–	–	–	6	9.1 %
36–40	–	–	1	1.5 %	–	–	–	–	–	–	1	1.5 %
41–45	1	1.5 %	–	–	–	–	1	1.5 %	–	–	2	3.0 %
46–50	1	1.5 %	–	–	–	–	–	–	–	–	1	1.5 %
51 and over	1	1.5 %	–	–	–	–	–	–	–	–	1	1.5 %
Total	44	66.7 %	17	25.8 %	3	4.5 %	1	1.5 %	1	1.5 %	66	100.0 %

**Fig. 1:**
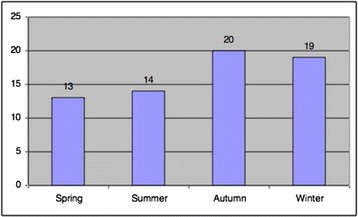
Distribution of cases according to seasons

**Fig. 2:**
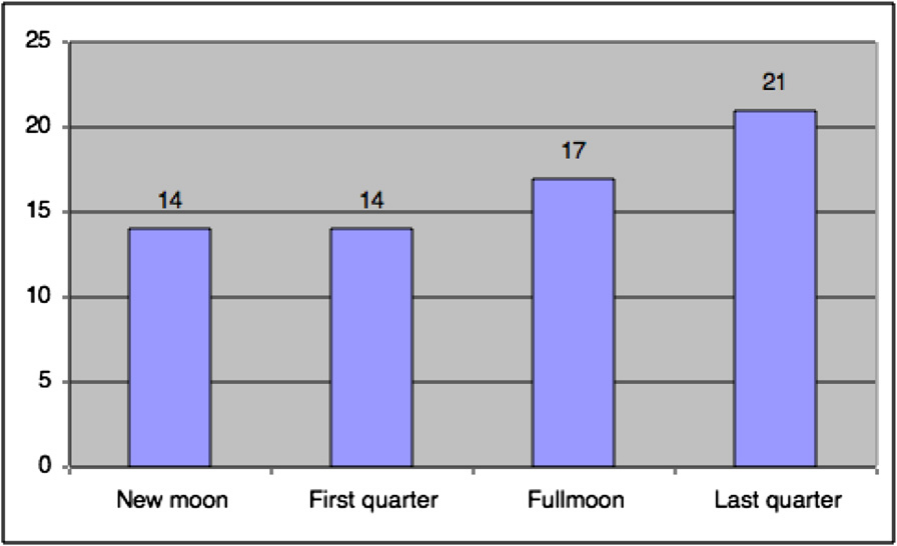
Distribution of cases according to lunar phases

The majority of the suicide deaths occurred in the months of July, September, October and December, with eight (12.1 %) cases in each. The fewest cases were seen in June, with only one (1.5 %) case. There were no statistically significant differences between month and age and between month and marital status when compared using a chi-square test (*p* > 0.05 for both, chi-square = 0.933 and 0.389, respectively). The distribution of the cases according to seasons is shown in Fig. 1, and the moon phases on the dates of the suicides are shown in Fig. 2. Again, there were no statistically significant differences between moon phase and age and between moon phase and marital status by chi-square test (*p* > 0.05 for both, chi-square = 0.583 and 0.433, respectively).

The marital status of the cases were as follows: 22 (33.3 %) were married in an official or religious way, 39 (59.1 %) were single and five (7.6 %) were divorced from their spouses. Of the married cases, three (4.5 %) were married as a fellow wife and 10 (15.2 %) were married through a bride exchange. Comparison of the age and marital status groups revealed statistically significant differences (*p* < 0.05 and chi-square = 0.012). One of the nine cases under the age of 15 (11.1 %) was married through bride exchange and a religious ceremony. Fourteen (21.21 %) of the married or divorced cases had children, 52 (78.8 %) of the cases had no children and none of the single cases had children. The number of children was one for three cases, two for six cases, three for one case, four for three cases and six for one case. Statistically significant differences were found when the cases were evaluated based on age group and number of children (*p* < 0.001 and chi-square = 0.000) (Table 2).

The professional status of the cases was as follows: 58 (87.9 %) were housewives, one (1.5 %) was a labourer and seven (10.6 %) were students. No statistically significant differences were found when the professional status of the cases was compared with their marital status (*p* > 0.05, chi-square = 0.168). However, there were statistically significant differences between professional status and age group (*p* < 0.05, chi-square = 0.018).

The economic status was as follows: very poor for 57 (86.4 %) cases, poor for two (3 %) and moderate for another two (3 %), good for four (6.1 %) and very good for one (1.5 %). There were no statistically significant differences between economic status and age and between economic status and marital status (*p* > 0.05 for both, chi-square = 0.865 and 0.679, respectively).

Five (7.6 %) of the cases under treatment for depression, and 12 (18.2 %) had made a previous suicide attempt. The women with a history of treatment for depression and those who had made a previous suicide attempt were considered as a single group. When this group was compared with the marital-status groups and separately with the age groups, no statistically significant differences were noted (*p* > 0.05 for both, chi square = 0.360 and 0.051, respectively).

The external examination of 12 (18.2 %) cases revealed traumatic signs related to violence such as ecchymosis, laceration and haematoma that occurred in different time periods. These traumatic signs were new in four cases (6.1 %). No statistically significant differences were found between the traumatic signs and marital status or between traumatic signs and age (*p* > 0.05 for both, chi-square = 0.150 and 0.692, respectively).

The scene of the incident was a house in 39 cases (59.1 %) and a barn in 16 cases (24.2 %). In 11 cases (16.7 %), the scene could not be determined. There were no statistically significant differences between the scene of the incident and age, between scene of incident and season and between scene of incident and lunar period (*p* > 0.05, chi square = 0.863, 0.124 and 0.664, respectively).

Suicide methods were as follows: hanging in 44 cases (66.7 %), firearm injury in 17 cases (25.8 %), insecticide intoxication in three cases (4.5 %), and jumping from a height in one case (1.5 %). One case was a 16-year-old who attempted a complex suicide by stabbing and firearm use. The distribution of the suicide methods based on age group is shown in Table 1. There were statistically significant differences between suicide methods and age. Among individuals under the age of 21, the most frequent suicide method was hanging (*p* < 0.05, chi-square = 0.032).

Another group consisted of two cases. One (1.5 %) was a dyadic death. A suicide note was found written on the body of the other case, who had died from hanging (complete, typical). When this group was compared with the marital-status groups and separately with age groups, no statistically significant differences were found (*p* > 0.05 for both, chi-square = 0.951 and 0.976, respectively).

## Discussion

In our study, it was determined that nearly half of the cases (45.45 %) were children under 18 years old and nine (13.6 %) were under 15 years old. Suicide frequency was not affected by lunar periods or seasonal changes, and most of the married cases (59 %) were fellow wives or married through bride exchange. Additionally, 78.8 % of the cases did not have any children and 86.4 % of the cases were financially troubled. The most common suicide method was hanging.

Because completed suicides (suicides that end in death) are seen more frequently among males in Turkey, it is notable that in Van the reverse is true. A total of 431 suicide and attempted suicide cases recorded from 2000 to 2003 in Van were assessed by Sancak from the Van Yüzüncü Yıl University Research Center for Women Studies. According to the reports of the provincial security directorate for incidents from the city centre and provincial gendarmerie command for incidents from towns or villages, 151 of 431 cases (80 females, 71 males) were completed suicides [18]. Similarly, in our study, we analysed the suicide deaths (completed suicides) in Van city centre from 2005 to 2011, and the number of female cases was higher than the number of male cases, with 66 females and 57 males out of a total of 123 suicide cases. It is also remarkable that the number of completed suicides from 2000 to 2003 (n = 151) was higher in the study by Sancak [18] when compared to our study, which involved six years of data on suicide deaths (n = 123). We attribute this result to the fact that our study involved suicide cases mainly in the city centre whereas Sancak’s study involved suicide cases in both the city centre and towns or villages.

Suicide is rare in childhood, and the incidence increases with age [16]. Adolescence is a high-risk period for suicide attempts [19]. In the study by Sancak, 80 female suicides occurred in Van in 2000–2003. Of these cases, 15 (18.7 %) were 15 years old and younger, 33 (41.3 %) were between 16 and 20 years old and only six (7.5 %) cases were 35 years old and older [18]. The percentages for these age groups in our study were 13.6 %, 45.5 % and 7.5 %, respectively. The findings indicate that female suicides increase in childhood and adolescence and are much less common among those over 35 years old. Due to the patriarchal culture of the Van area, girls are married at a young age, cannot have an occupation and are not allowed to work [20]. Sancak also reported that the high rate of childhood and adolescent female suicide could be the result of the closed structure of the society, which means continuation of large families and forcing girls to stay at home, to drop out of school and to marry at a very young age [18]. The dramatic decrease in suicide rates as age increases is thought to be due to the respect for elderly people in the cultural and traditional family structure of Van. In other words, women who had no right to speak when they were young become more respected and recognized in the family and society as they get older.

The cause, origin and region of the death cases show seasonal variations in almost every country all over the world [21–23]. According to a study that evaluated homicides and suicides in India, deaths from homicide and suicide were most common in summer, suicides peaked in April and May and homicides peaked in October [24]. A study from Japan reported that suicides reached peaks in April and the fall, whereas the seasonal effect was minimal for deaths resulting from cancer or homicide [5]. Ramadan is a lunar calendar month in which Muslims refrain from eating, drinking and sexual intercourse from dawn to sunset, and it has been reported that, although there is a significant decline in traumatic deaths during Ramadan, the incidence of suicides increases, particularly among females [25]. In our study, 32 suicides (48.48 %) occurred in July, September, October and December. There were no statistically significant differences between suicide periods and moon phases (*p* > 0.05). Some previous studies also found no correlation between female and male suicides and moon phase [26, 27].

Depression; being single, divorced, or widowed; young age; considering an abortion; and low educational and socioeconomic status are suicide risk factors [28]. According to a study performed in Hungary, a typical female who attempts suicide could be characterized as follows: retired or economically inactive, widowed or divorced, or having depression in her personal history [29]. Additionally, severe conflicts caused by a troubled marriage are known to increase suicide tendency [30].

According to a study conducted in Adıyaman, a province in the eastern part of Turkey, 41.72 % of the females who committed suicide were married [31]. In our study, 33.3 % (n = 22) of the cases were married in an official or religious way, and five cases (7.6 %) were divorced. It is also notable that 13 (59 %) of the married cases were fellow wives or married through bride exchange.

In addition, it is known that having a child decreases suicide attempts and death ideation [32, 33]. In our study, only 14 cases (21.2 %) had a child, whereas almost 80 % of the cases were females who did not have any children. It is also interesting that 39 (75 %) out of 52 females who did not have children were single, and the comparison of age vs. marital status and age vs. number of children revealed statistically significant results.

Migration to the city centre has increased due to evacuation of villages in Van because of terrorism. The differences between village and city life make the adaptation of emigrating people more difficult. Families who lived by breeding livestock in their villages were left unemployed in the city centre and therefore had severe financial problems [18]. Low socioeconomic status, being retired and being economically inactive are among suicidal risk factors for women [28, 29]. In our study, only one (1.5 %) of the cases was employed, 87.9 % were housewives and 10.6 % were students, for a total of 98.5 % who did not have financial independence. Thus, the economic status was very poor for 86.4 % of the cases in our study and poor for 3 %.

The relationship between violence against women and suicide is well known in countries with moderate and low socioeconomic status [14]. Violence affects women in more negative ways than it does men [34]. In our study, the presence of new or old traumatic findings in 12 cases (18.2 %) indicated that the women had probably been exposed to violence.

Suicidal thoughts can be related to serious depression, being abused during childhood, early onset of a major depressive disorder or poor living conditions [35]. The assessment of suicide risk in patients diagnosed with depression is a major step toward precautionary measures [36]. The history of a previous suicide attempt increases the possibility of a successful suicide [16]. In our study, five (7.6 %) cases were undergoing depression treatment, and 12 (18.2 %) had previously attempted suicide. The fact that seven cases (10.6 %) were students indicated the importance of psychological counselling and guidance on addressing problems for students.

In a study from Persia, the percentage of suicides committed at home was 75 % [37]. In our study, the predominant suicide location was a house (59.1 %) or a barn (24.2 %), which is used as an extension of the house in Anatolian culture, and constituted 83.33 % of the incidents.

The lethality of the methods that were preferred in incomplete female suicides is generally lower than that of the methods used in successful suicides, the latter are more traumatic. Hanging is the most frequently seen method in developing countries, whereas firearm use is more frequent in developed countries [38, 39]. According to Turkish Statistical Institute data for 2002–2009, of 21,752 suicide cases in Turkey, 14,012 (35.6 %) were females, and 42.9 % of them committed suicide by hanging [40]. In a study conducted in Kahramanmaras City, Turkey from 1992 to 2002, of 128 suicide incidents, 49 cases were females, and the most frequently used method was hanging [41]. Concordantly, in our study, the most frequently seen suicide method was hanging, for 44 cases (66.7 %).

Sometimes, suicide victims leave suicide notes to their acquaintances or the people who will find them. The notes can be written on paper, on materials similar to paper or, extraordinarily, on the suicide victim’s own body [42]. As mentioned above, there was a suicide note on the body of one of our cases.

The limitations of this study include the kind of autopsies reviewed; autopsies of cases that occurred in the city centre were reviewed, but the autopsy reports belonging to the county prosecution offices were not included. Therefore, an analysis of these county cases could not be performed. In addition, because the autopsy reports that constituted the study material did not contain the cause of suicide or the victim’s educational background, these two parameters were not included.

## Conclusions

In contrast to the general population of Turkey, a higher incidence of female suicides in Van indicates a need for closer attention to women’s issues in this region.

According to our results, approximately half of the cases were children, 90 % were housewives and 10 % were students. It is known that housewives and students have no economic freedom. Furthermore, the economic status of 90 % of the cases was classified as poor or very poor. Closer and more efficient attention by the Ministry of Family and Social Policies to families with financial difficulties can contribute to the prevention of suicides caused by such problems. Supporting home-based businesses can be helpful by providing additional income for housewives. Additionally, school counsellors should maintain close follow-up of and give necessary guidance to students exposed to violence, abuse or forced marriages. Providing medical follow-up, psychological consultation and family therapy can be helpful for children, adolescents and women who have a history of depression, suicide attempt or suicidal thoughts. Families who allow underage children to marry instead of continuing their education should be prosecuted as criminals, and the child victim should be taken into foster care for the continuation of her education.

The groom, parents and other family members who allow the underage marriage, the chaplain who does not have the right to perform such marriage ceremonies without a civil marriage and the village chief and other officials who do not report the situation should face heavy criminal penalties. The state should not ignore men who have a fellow wife or who marry through bride exchange without the women’s consent, and criminal prosecution should be undertaken when a non-consensual act has occurred.

Traumatic findings indicating possible exposure to violence were found in the autopsies of some cases in our study. Violence against women should be discussed at all educational levels in order to promote awareness. Religious and political leaders, artists, athletes and any person who has a prominent role in society should take part in the awareness campaign. Another problem to address is the belief by most victims that they will not receive protection if they report the violence to legal authorities; in fact, they believe they will be subjected to more violence if they do so. Thus, domestic violence complaints in police stations and courthouses should be processed in an easy and straightforward manner so as to foster trust in the legal system.

Moreover, encouraging individual disarmament and prevention of having firearms in the home are also important to prevent the use of firearms not only for suicidal reasons but also for any other kind of violence.
